# Predicting Early Outcomes of Prostatic Artery Embolization Using *n*-Butyl Cyanoacrylate Liquid Embolic Agent: A Machine Learning Study

**DOI:** 10.3390/diagnostics15111351

**Published:** 2025-05-28

**Authors:** Burak Berksu Ozkara, David Bamshad, Ramita Gowda, Mert Karabacak, Vivian Bishay, Kirema Garcia-Reyes, Ardeshir R. Rastinehad, Dan Shilo, Aaron Fischman

**Affiliations:** 1 Department of Diagnostic, Molecular and Interventional Radiology, Icahn School of Medicine at Mount Sinai, New York, NY 10029, USA; 2 School of Medicine, St. George’s University, True Blue, Grenada; 3 Department of Neurosurgery, Mount Sinai Health System, New York, NY 10029, USA; 4 Department of Urology, Lenox Hill Hospital, New York, NY 10075, USA

**Keywords:** prostate artery embolization, machine learning, IPSS, artificial intelligence

## Abstract

**Background/Objectives**: Prostatic artery embolization (PAE) has been increasingly recognized, especially with recent progress in embolization techniques for the management of lower urinary tract symptoms due to benign prostatic hyperplasia. Nevertheless, a proportion of patients undergoing PAE fail to demonstrate clinical improvement. Machine learning models have the potential to provide valuable prognostic insights for patients undergoing PAE. **Methods**: A retrospective cohort study was performed utilizing a modified prior-data fitted network architecture to predict short-term (7 weeks) favorable outcomes, defined as a reduction greater than 9 points in the International Prostate Symptom Score (IPSS), in patients who underwent PAE with *n*BCA glue. Patients were stratified into two groups based on the median IPSS reduction value, and a binary classification model was developed to predict the outcome of interest. The model was developed using clinical tabular data, including both pre-procedural and intra-procedural variables. SHapley Additive ExPlanations (SHAP) were used to uncover the relative importance of features. **Results**: The final cohort included 109 patients. The model achieved an accuracy of 0.676, an MCC of 0.363, a precision of 0.666, a recall of 0.856, an F1-score of 0.731, and a Brier score of 0.203, with an AUPRC of 0.851 and an AUROC of 0.821. SHAP analysis identified pre-PAE IPSS, prior therapy, right embolization volume, preoperative quality of life, and age as the top five most influential features. **Conclusions**: Our model showed promising discrimination and calibration in predicting early outcomes of PAE with *n*BCA glue, highlighting the potential of precision medicine to deliver interpretable, individualized risk assessments.

## 1. Introduction

Prostatic artery embolization (PAE) is gaining prominence, particularly with recent advancements in embolization techniques for managing lower urinary tract symptoms associated with benign prostatic hyperplasia [1,2]. However, a subset of patients treated with PAE do not experience any improvement, and a notable proportion faces symptom recurrence [3,4,5]. Carnevale et al. reported symptom recurrence in 23% of patients at a median follow-up of 72 months, while Xu et al. observed cumulative recurrence rates of 6.8%, 12.7%, and 60.4% after 1, 2, and 5 years, respectively [3,4].

Several novel embolic agents, such as ethylene–vinyl alcohol copolymers, shear-thinning hydrogels, and on-demand degradable microspheres, are being developed to improve precision and safety in embolization procedures, with *n*-butyl cyanoacrylate (*n*BCA) emerging as one of the promising options in this evolving landscape. The use of *n*BCA glue for PAE represents a potentially significant advancement as an alternative to embolization with traditional microspheres, as demonstrated in recent studies showing improvements in patient outcome metrics such as the International Prostate Symptom Score (IPSS) and quality of life (QoL), along with potentially decreased recanalization rates [6,7,8]. While technical advancements are crucial for improving patient outcomes, optimizing patient selection through advanced interpretable machine learning (ML)/artificial intelligence (AI) models using pre-procedural data may also play a critical role, especially considering the risks inherent to any medical procedure [9,10]. With refinements and further improvements, such models may help determine whether to perform the procedure, foster informed discussions with patients about likely outcomes, and serve as a quality assessment tool when results fall below expectations [11].

The literature on the prognostic prediction of PAE outcomes using ML/AI is extremely limited, with most studies focusing on identifying individual parameters that influence outcomes [2,12,13,14]. While revealing singular parameters affecting outcomes is essential, by analyzing the combined effects of multiple variables and their interactions, ML/AI models may uncover hidden patterns and capture complex relationships, potentially aiding in prognostication and selecting suitable candidate patients. Martin et al. explored MRI radiomics but unfortunately provided limited benefit, while Vigneswaran et al. successfully utilized tabular pre-treatment data and applied traditional regression-based algorithms in their important study, such as linear regression, to predict the baseline IPSS and the change in IPSS 1 year after PAE [2,15].

In our study, we employed TabPFN, an advanced and novel ML model based on a modified prior-data fitted network (PFN) architecture, to predict IPSS reduction at a 6-week follow-up appointment of PAE performed with *n*BCA using pre-procedural and intra-procedural tabular data. The model was designed for binary classification of the outcome, distinguishing between favorable (IPSS reduction > 9) and unfavorable results. Unlike traditional techniques, our model incorporates interpretable tools, offering actionable insights into feature importance on outcomes. However, this study did not involve computer vision or imaging-derived features, focusing instead on clinical and procedural tabular data. All code has been made publicly available to enhance transparency and foster collaboration.

## 2. Materials and Methods

This retrospective cohort study used ML to predict a short-term (7 weeks) favorable outcome ([Pre-IPSS] − [Post-IPSS] = IPSS reduction > 9) in patients who underwent PAE. Patients were divided into two groups based on the median value and a binary classifier was created to predict the outcome of interest. Our study followed the Transparent Reporting of Multivariable Prediction Models for Individual Prognosis or Diagnosis–Artificial Intelligence statement [16].

### 2.1. Patient Selection

**Figure 1:**
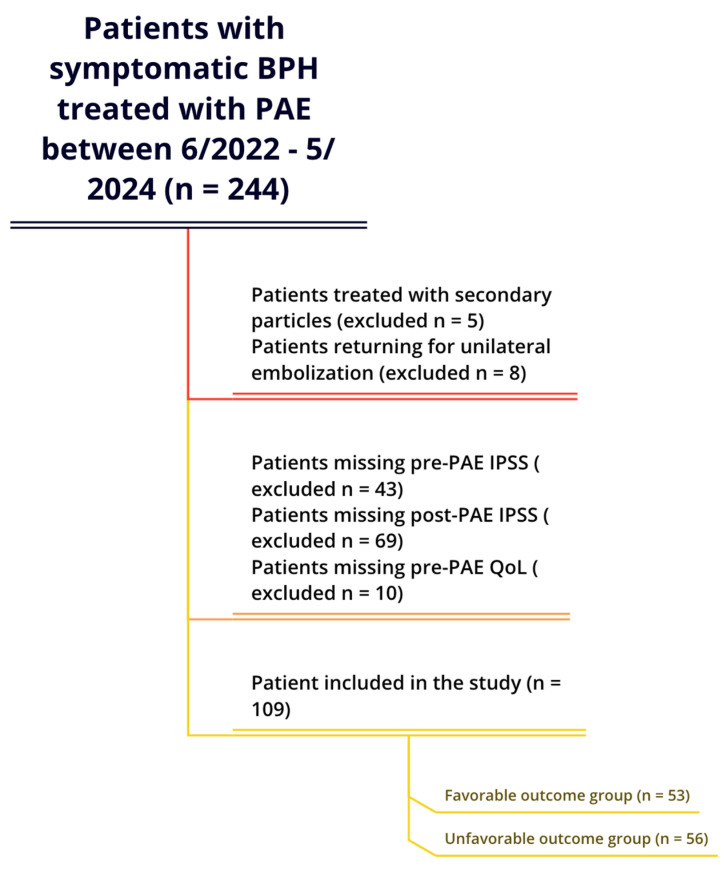
Patient selection.

This study used a patient cohort from a previous publication by our group [8]. A retrospective review was conducted on 244 consecutive patients who underwent PAE with *n*BCA and ethiodized oil from June 2022 to May 2024 (Figure 1). The study excluded patients who underwent secondary microsphere embolization (*n* = 5) or returned for contralateral embolization after initial unilateral embolization (*n* = 8), due to potential confounding effects. In addition, 43 patients missing pre-PAE IPSS, 69 patients missing post-PAE IPSS, and 10 patients missing pre-PAE QoL were excluded from the current study. The final cohort included 109 patients, with data sourced from a Health Insurance Portability and Accountability Act-compliant database under a retrospective protocol approved by the institutional review board at Mount Sinai Hospital (Study number: 21-01756). Due to this study’s retrospective nature, individual informed consent was not required.

### 2.2. Data Collection

The severity of clinical manifestations was evaluated using the 7-item IPSS and the single-item QoL scale, following the AUA Practice Guidelines Committee recommendations [17]. Baseline data were collected for age, pre-PAE IPSS and QoL, uroflowmetry parameters (Qmax and Qavg), prostate gland volume (PGV), pre-operative post-void residual (PVR), pre-operative hematuria, prior therapies, and history of intermittent catheterization or indwelling catheter use. Procedural details, including right and left embolization volumes, the dilution ratio of the embolization material, and the treatment approach (bilateral glue embolization versus unilateral glue embolization with contralateral coil embolization), were documented. The primary outcome, IPSS reduction, was assessed at the 6-week follow-up appointment post PAE. The details of the PAE procedure are explained in the prior study [8].

### 2.3. Data Preprocessing

To minimize exclusion bias, imputation methods were applied to handle missing data. Among the continuous variables, six had at least one missing value. Variables with over 30% missing data (Qavg, Qmax, and PVR) were excluded. For the remaining variables, the k-nearest neighbor (kNN) algorithm (k = 5) was used to impute missing values by drawing on data from the entire dataset [18]. The kNN method fills in missing values using data from the five most similar cases. For categorical variables, two had missing values, but none were excluded since no variable had more than 30% missing data. Missing values in categorical variables were imputed using the mode. After these steps, all variables were included in the model without feature selection.

### 2.4. Model Development and Assessment

A modified PFN architecture (TabPFN) was employed for model development. The model was developed to perform binary classification, differentiating between favorable outcomes (IPSS reduction > 9) and unfavorable outcomes (IPSS reduction ≤ 9). The >9-point threshold was chosen based on the median IPSS reduction in our cohort to enable a balanced binary classification for model training. TabPFN leverages a meta-learning framework to adapt to novel, unseen data by drawing on insights from various dataset. Prior-data fitted networks are pre-trained on synthetic datasets to approximate Bayesian inference for real-world scenarios, enabling TabPFN to recognize complex patterns in tabular data and adapt well to new datasets, making it especially valuable for small to medium-sized tabular datasets due to its ability to generalize without extensive hyperparameter tuning [19].

Model performance assessment was performed with a 10-repeat, 10-fold stratified cross-validation technique. The dataset was divided into ten nearly equal parts for each repetition with a different random split, ensuring that the outcome class proportions remained consistent across all parts. For each fold in every repetition, the data were divided into a training subset (70% of the total data), a validation subset (10%), and a test set (20%). The validation sets were used for sigmoid calibration to tune predicted risks to observed outcomes. Predictions and probability estimates were generated by the calibrated TabPFN model on each test fold across the ten cross-validation repetitions. Results from all folds and repeats were combined to assess overall performance. Implementing ten repeats enhances the robustness of performance evaluations, minimizes the influence of particular random splits, reduces the risk of overfitting to specific data partitions, and offers a more thorough analysis of model stability. Overall, cross-validation provides a dependable evaluation of the model’s generalizable predictive capabilities [11].

Model performance was visually assessed using a receiver operating characteristic (ROC) curve, a precision–recall curve (PRC), and a calibration plot to evaluate the alignment between predicted probabilities and actual outcomes. Furthermore, the area under the ROC curve (AUROC), the area under the PRC (AUPRC), the Brier score, precision, recall, F1-score, and Matthews Correlation Coefficient (MCC) were calculated. For each metric, a 95% confidence interval (CI) was calculated using bootstrapping with 1000 resampled datasets, determined by the 2.5th and 97.5th percentiles of the bootstrapped metric mean values.

To enhance interpretability, SHapley Additive ExPlanations (SHAP) were used to uncover the relative importance of features. The SHAP plot ranked the features in descending order, displaying the most impactful feature at the top. The study’s GitHub repository (https://github.com/mertkarabacak/PAE-ML [accessed on 22 May 2025]) provides access to the model code, ensuring complete transparency.

## 3. Results

As shown on Figure 1, the final cohort included 109 patients in our study. Table 1 presents the patient characteristics for the unfavorable outcome group, the favorable outcome group, and the overall cohort. The mean short-term follow-up period was 7 weeks. All patients underwent PAE with *n*BCA, and all were technically successful. The favorable outcome group (IPSS reduction > 9) consisted of 53 patients, whereas the unfavorable outcome group (IPSS reduction ≤ 9) included 56 patients. The median age was 73.5 (interquartile range [IQR] = 13.5) years in the unfavorable outcome group, 70 (IQR = 12.5) in the favorable outcome group, and 72 (IQR = 13) in the entire cohort.

The mean pre-PAE IPSS was 16.1 (standard deviation [SD] = 5) in the unfavorable outcome group, 24.5 (SD = 5.2) in the favorable outcome group, and 20.2 (SD = 6.6) across all participants. The median pre-PAE QoL score was 4 (IQR = 1) in the unfavorable outcome group, 5 (IQR = 1.5) in the favorable outcome group, and 4 (IQR = 2) in the overall cohort. The median pre-PAE PGV was 86.5 mL (IQR = 94) in the unfavorable outcome group, 135 mL (IQR = 106.85) in the favorable outcome group, and 109 mL (IQR = 117.6) in the overall cohort. History of prior therapy was reported in 25% (*n* = 14) of the unfavorable outcome group and 7.5% (*n* = 4) of the favorable outcome group. Unilateral glue embolization with contralateral coil embolization was performed in 7.1% (*n* = 4) of the unfavorable outcome group and 1.9% (*n* = 1) of the favorable outcome group, with the remaining patients receiving bilateral glue embolization. The median IPSS reduction was 4.5 (IQR = 5) in the unfavorable outcome group, 16.5 (IQR = 7) in the favorable outcome group, and 9 (IQR = 11.8) in the full cohort.

**Figure 2:**
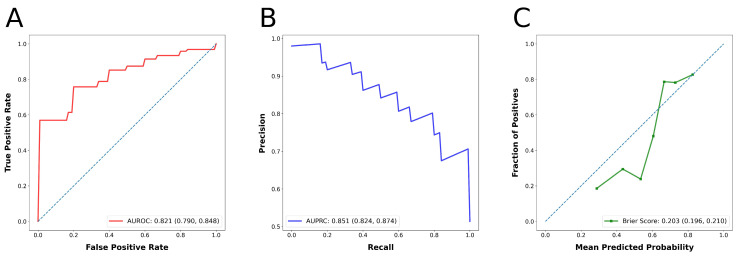
The receiver operating characteristic curve (**A**), precision–recall curve (**B**), and calibration curve (**C**) for the model’s performance.

Table 2 summarizes the model evaluation results. The predictive capabilities of the model were demonstrated by an accuracy of 0.676 (95% CI: 0.647–0.705), an MCC of 0.363 (95% CI: 0.302–0.423), a precision of 0.666 (95% CI: 0.640–0.698), a recall of 0.856 (95% CI: 0.825–0.885), an F1-score of 0.731 (95% CI: 0.709–0.752), and a Brier score of 0.203 (95% CI: 0.196–0.210). The AUPRC was 0.851 (95% CI: 0.824–0.874), while the AUROC was 0.821 (95% CI: 0.790–0.848), reflecting excellent discriminatory ability [20]. The ROC curve, PRC, and calibration curve for the model’s performance are shown in Figure 2A–C, respectively.

**Figure 3:**
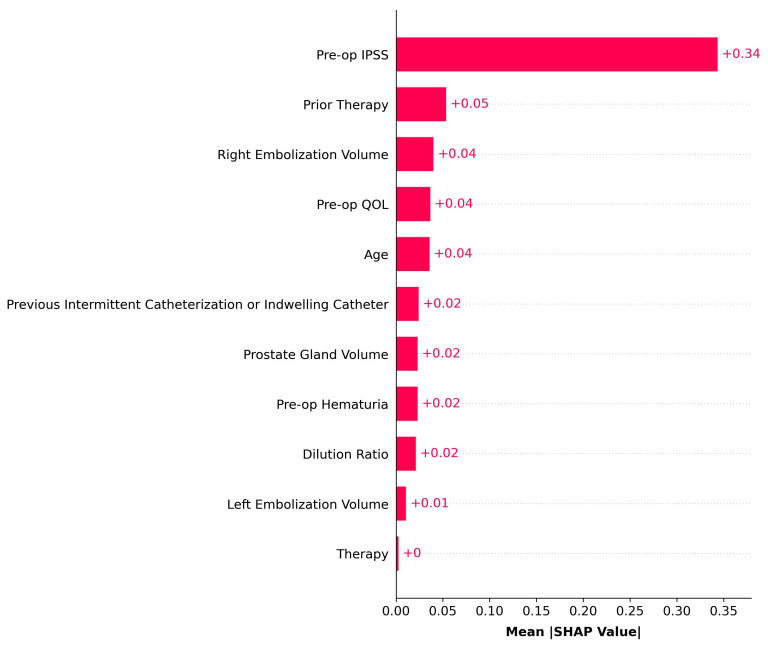
SHapley Additive exPlanations. IPSS = International Prostate Symptom Score; QoL = quality of life.

Figure 3 illustrates the relative feature importance based on SHAP values, revealing pre-PAE IPSS, prior therapy, right embolization volume, pre-op QoL, and age as the top five most important features.

## 4. Discussion

Although trial results will be critical for determining the efficacy of *n*BCA glue in PAE and improving patient selection, reliable ML/AI-based prognostic tools can also play an important role in facilitating informed discussions with patients about their expected outcomes. Our study demonstrates the potential of ML/AI models to improve prognostic predictions for patients undergoing PAE with *n*BCA glue by developing a valuable tool for predicting the early favorable outcome. Our model achieved an AUROC of 0.821 and a Brier score of 0.203, demonstrating promising discriminatory ability and calibration for predicting the early favorable outcome. These results highlight the potential of ML/AI to enable individualized prognostication for PAE with *n*BCA glue by leveraging key clinical parameters. To the best of our knowledge, this is the first prognostic ML model specifically developed to predict IPSS reduction outcomes in PAE procedures using *n*BCA glue.

A key strength of our approach lies in the inclusion of the SHAP feature importance plot, which offers a detailed overview of how critical features influence predicted outcomes across the entire dataset, thereby revealing the underlying behavior of the model. This approach provides a level of transparency that is not seen in previous studies, allowing for a deeper understanding of the factors driving each prediction [11]. This feature enhances the model’s interpretability and bolsters its credibility, helping researchers to evaluate the key influences on predictions.

In our study, global SHAP analysis revealed that the five most influential features in predicting outcomes were pre-PAE IPSS, prior therapy, right embolization volume, pre-PAE QoL, and age. Bilhim et al. identified older age and higher pre-PAE IPSS as predictors of clinical failure following PAE, findings that are consistent with our study, where these features also emerged as key features in our model’s predictions [14]. Given that higher pre-PAE IPSS predicts clinical failure following PAE, it suggests that baseline QoL, often correlated with IPSS, may predict clinical success, emphasizing its role as a key feature in our model [21]. Right embolization volume was another critical feature in our study. Although its significance may not be immediately apparent, one possible explanation is that Zhang et al. found that 67.3% of their study population had a prostate primarily supplied by a unilateral prostatic artery, and in our cohort, the right side may be the dominant supply in some patients, which could have influenced the outcomes and predictions [22]. The inclusion of such procedural factors highlights the potential utility of ML models not only for pre-procedural prognostication but also as tools to inform and adapt intra-procedural decision-making in real time.

As stated earlier, to our knowledge, this is the first study exploring a prognostic ML model to predict outcomes in PAE procedures using *n*BCA glue. Meanwhile, Vigneswaran et al. recently published an impressive study on predicting exact IPSS changes 1 year after PAE (the exact embolization techniques are not mentioned) using traditional models such as linear regression and random forests [2]. It is worth noting the differences between the two studies. Firstly, our study focused on predicting binary outcomes based on IPSS change, categorizing patients into those with favorable and unfavorable outcomes rather than predicting the exact change in IPSS. Predicting binary outcomes may simplify the interpretation of results for clinicians, providing straightforward classifications that are easier to apply in practice, similar to urologic literature, which defines clinical success as an improvement in IPSS of >25%, a binary outcome structure [23]. Meanwhile, detailed predictions such as exact IPSS changes may also be beneficial for clinical decision-making, as even a modest advancement in IPSS might be deemed adequate to prefer PAE over more invasive techniques [12]. Moreover, we predicted early outcomes, in contrast to their 1-year follow-up, where long-term outcomes may be more clinically meaningful. To accurately evaluate the lasting benefits of PAE, a prolonged follow-up period is necessary, as clinical improvements may emerge gradually. Furthermore, we implemented sophisticated imputation methods for missing data, in contrast to including only patients with complete records, which could lead to potential bias [24]. Moreover, we employed TabPFN, an advanced meta-learning model, which enhances model adaptability and generalization to unseen data, providing a robust approach for predicting patient outcomes, particularly when dealing with small datasets [25]. Another important advantage of our study is using the advanced explainability method SHAP. SHAP values provide a clear and quantitative understanding of how each feature influences the model’s predictions by showing their importance values. This approach allows for increased transparency, making it easier for clinicians to understand the rationale behind the predictions. This is especially important, as it is well known that black box models cause interpretability issues, making clinicians hesitant [10]. By identifying the most impactful features, SHAP analysis not only improves model interpretability but also provides actionable insights that may help guide clinical decision-making, ensuring that the model’s outputs are both reliable and clinically relevant. While the two studies have differences, we believe both are important foundational studies, serving as pioneering contributions to the field.

Despite our rigorous methods, our study has limitations. Firstly, our study has the inherent limitations of retrospective research. The modest sample size limits the generalizability of our findings. The lack of an independent test set further restricts our ability to evaluate the model’s generalizability. Furthermore, the data were sourced from a high-volume tertiary center, which may not reflect the broader range of clinical settings where PAEs are performed. Another limitation of our study is that we focused on short-term outcomes, which may not necessarily predict long-term outcomes. The clinical effects of PAE may evolve over time, and a longer follow-up period would be needed to capture sustained efficacy [26]. Also, using kNN for imputing missing data has limitations when handling non-random missingness, which could potentially introduce bias. Moreover, the distribution of outcomes reflects a methodological decision to dichotomize patients based on the median IPSS reduction, enabling a balanced dataset for binary classification modeling. Consequently, patients with an IPSS reduction of ≤9 were labeled as having “unfavorable” outcomes purely for statistical modeling purposes; this categorization does not imply procedural failure in a clinical sense. Rather, it serves as a pragmatic framework to facilitate the development and validation of machine learning models. Another limitation of this study is the strong influence of baseline IPSS on model performance. Although the model integrated a variety of clinical and procedural features, its predictive capacity may still be partially driven by the baseline symptom burden, which differed significantly between outcome groups. This may limit the incremental benefit of using complex models over simpler heuristics in certain scenarios. Future work using continuous outcome measures or longer follow-up intervals may help clarify the added value of ML/AI approaches beyond dominant single predictors. It is important to note that although certain variables show prognostic relationships with outcomes, these associations should not be interpreted as causal without further investigation. The model predicts outcomes based on patterns and correlations, not on establishing causality. Therefore, model outputs should be regarded as prognostic predictions rather than confirmations of causal mechanisms or treatment effectiveness. This model should not be used in clinical settings.

## 5. Conclusions

In conclusion, our ML approach demonstrated promising discrimination and calibration in predicting early outcomes for PAE with *n*BCA glue. By converting predictive modeling into transparent and interpretable risk assessments, our method highlights the potential of precision medicine to provide detailed prognostic insights for PAEs. However, the current model should be regarded as a proof-of-concept and a research tool for risk stratification rather than a clinically deployable decision-support system. Larger multicenter datasets, external validation, and long-term follow-up are essential next steps to assess generalizability. Prospective studies, the evaluation of real-world clinical impact, and model calibration across diverse patient populations will be necessary before such a tool can be safely and effectively integrated into clinical decision-making.

## Figures and Tables

**Table 1:** Patient characteristics. IQR = interquartile range, SD = standard deviation, PAE = prostate artery embolization, IPSS = International Prostate Symptom Score, QoL = quality of life, Qmax = maximum flow rate, Qavg = average flow rate, PGV = prostate gland volume, and PVR = post-void residual.

Variables		Unfavorable Outcome Group (*n* = 56)	Favorable Outcome Group (*n* = 53)	Combined (*n* = 109)
Baseline Characteristics				
Age	Median (IQR), *n*	73.5 (13.5), 56	70 (12.5), 53	72 (13), 109
	Mean ± SD, *n*	73.7 ± 9.7, 56	70 ± 8, 53	71.9 ± 9.1, 109
Pre-PAE IPSS	Median (IQR), *n*	16 (7), 56	25 (8.5), 53	20 (9.5), 109
	Mean ± SD, *n*	16.1 ± 5, 56	24.5 ± 5.2, 53	20.2 ± 6.6, 109
Pre-PAE QoL	Median (IQR), *n*	4 (1), 56	5 (1.5), 53	4 (2), 109
	Mean ± SD, *n*	3.6 ± 1.2, 56	4.2 ± 1.1, 53	3.9 ± 1.2, 109
Pre-PAE Qmax	Median (IQR), *n*	7 (5.5), 33	6.9 (4.4), 29	7 (4), 62
	Mean ± SD, *n*	8.7 ± 6.9, 33	12.7 ± 33.5, 29	10.6 ± 23.3, 62
Pre-PAE Qavg	Median (IQR), *n*	4.05 (4), 6	4.5 (1.55), 12	4.4 (1.9), 18
	Mean ± SD, *n*	5 ± 2, 6	4.5 ± 1.8, 12	4.6 ± 1.8, 18
Pre-PAE PGV	Median (IQR), *n*	86.5 (94), 38	135 (106.85), 40	109 (117.6), 78
	Mean ± SD, *n*	118.9 ± 85.6, 38	143.8 ± 82.1, 40	131.7 ± 84.2, 78
Pre-PAE PVR	Median (IQR), *n*	133.2 (181.5), 20	100 (257.5), 21	120 (235.5), 41
	Mean ± SD, *n*	217 ± 266, 20	167.5 ± 166.7, 21	191.6 ± 219.4, 41
Pre-PAE Hematuria	Yes	20 (35.7%)	21 (39.6%)	41 (37.6%)
	No	27 (48.2%)	29 (54.7%)	56 (51.4%)
	N/A	9 (16.1%)	3 (5.7%)	12 (11.0%)
Prior Therapy	Yes	14 (25%)	4 (7.5%)	18 (16.5%)
	No	42 (75%)	49 (92.5%)	91 (83.5%)
	N/A	0 (0%)	0 (0%)	0 (0%)
Prior Catheterization	Yes	10 (17.9%)	11 (20.8%)	21 (19.3%)
	No	45 (80.3%)	42 (79.2%)	87 (79.8%)
	N/A	1 (1.8%)	0 (0%)	1 (0.9%)
Procedural Characteristics				
Left Embolization Volume	Median (IQR), *n*	1 (0.3), 49	1 (0.4), 49	1 (0.3), 98
	Mean ± SD, *n*	0.91 ± 0.34, 49	0.90 ± 0.37, 49	0.90 ± 0.35, 98
Right Embolization Volume	Median (IQR), *n*	1 (0.3), 49	1 (0.4), 48	1 (0.3), 97
	Mean ± SD, *n*	0.98 ± 0.47, 49	0.91 ± 0.4, 48	0.95 ± 0.43, 97
Dilution Ratio	Median (IQR), *n*	0.1 (0), 54	0.1 (0), 52	0.1 (0), 106
	Mean ± SD, *n*	0.098 ± 0.006, 54	0.097 ± 0.007, 52	0.098 ± 0.007, 106
Treatment Approach	BGE	52 (92.9%)	52 (98.1%)	104 (95.4%)
	UGE with CCE	4 (7.1%)	1 (1.9%)	5 (4.6%)
Outcome				
IPSS Reduction	Median (IQR), *n*	4.5 (5), 56	16.5 (7), 53	9 (11.8), 109
	Mean ± SD, *n*	3.7 ± 4.6, 56	17.1 ± 5, 53	10.2 ± 8.2, 109

**Table 2:** Model evaluation results. AUROC = area under the receiver operating characteristic curve; AUPRC = area under the precision–recall curve.

Performance Metric	Metric Value (95% Confidence Interval)
Precision	0.666 (0.640, 0.698)
Recall	0.856 (0.825, 0.885)
F1 Score	0.731 (0.709, 0.752)
Accuracy	0.676 (0.647, 0.705)
Matthews Correlation Coefficient	0.363 (0.302, 0.423)
AUROC	0.821 (0.790, 0.848)
AUPRC	0.851 (0.824, 0.874)
Brier Score	0.203 (0.196, 0.210)

## Data Availability

The data are unavailable due to privacy and ethical restrictions.

## Abbreviations

The following abbreviations are used in this manuscript:

PAE	Prostatic artery embolization
*n*BCA	*n*-butyl cyanoacrylate
IPSS	International Prostate Symptom Score
QoL	Quality of life
ML	Machine learning
AI	Artificial intelligence
PFN	Prior-data fitted network
Qmax	Maximum flow rate
Qavg	Average flow rate
PGV	Prostate gland volume
PVR	Post-void residual
kNN	K-nearest neighbor
ROC	Receiver operating characteristic
PRC	Precision–recall curve
AUROC	Area under the ROC curve
AUPRC	Area under the PRC
MCC	Matthews Correlation Coefficient
CI	Confidence interval
SHAP	SHapley Additive ExPlanations
IQR	Interquartile range
SD	Standard deviation
